# Finding the limits of deep learning clinical sensitivity with fractional anisotropy (FA) microstructure maps

**DOI:** 10.3389/fninf.2024.1415085

**Published:** 2024-06-12

**Authors:** Marta Gaviraghi, Antonio Ricciardi, Fulvia Palesi, Wallace Brownlee, Paolo Vitali, Ferran Prados, Baris Kanber, Claudia A. M. Gandini Wheeler-Kingshott

**Affiliations:** ^1^Department of Brain and Behavioral Sciences, University of Pavia, Pavia, Italy; ^2^NMR Research Unit, Department of Neuroinflammation, Queen Square Multiple Sclerosis Centre, UCL Queen Square Institute of Neurology, University College London, London, United Kingdom; ^3^Department of Radiology, IRCCS Policlinico San Donato, Milan, Italy; ^4^Department of Biomedical Sciences for Health, Universitá degli Studi di Milano, Milan, Italy; ^5^Department of Medical Physics and Biomedical Engineering, Centre for Medical Image Computing, University College London, London, United Kingdom; ^6^E-Health Center, Universitat Oberta de Catalunya, Barcelona, Spain; ^7^Brain Connectivity Centre, IRCCS Mondino Foundation, Pavia, Italy

**Keywords:** diffusion MRI, deep learning, fast sequence, clinical sensitivity, temporal lobe epilepsy, multiple sclerosis, fractional anisotropy

## Abstract

**Background:**

Quantitative maps obtained with diffusion weighted (DW) imaging, such as fractional anisotropy (FA) –calculated by fitting the diffusion tensor (DT) model to the data,—are very useful to study neurological diseases. To fit this map accurately, acquisition times of the order of several minutes are needed because many noncollinear DW volumes must be acquired to reduce directional biases. Deep learning (DL) can be used to reduce acquisition times by reducing the number of DW volumes. We already developed a DL network named “one-minute FA,” which uses 10 DW volumes to obtain FA maps, maintaining the same characteristics and clinical sensitivity of the FA maps calculated with the standard method using more volumes. Recent publications have indicated that it is possible to train DL networks and obtain FA maps even with 4 DW input volumes, far less than the minimum number of directions for the mathematical estimation of the DT.

**Methods:**

Here we investigated the impact of reducing the number of DW input volumes to 4 or 7, and evaluated the performance and clinical sensitivity of the corresponding DL networks trained to calculate FA, while comparing results also with those using our one-minute FA. Each network training was performed on the human connectome project open-access dataset that has a high resolution and many DW volumes, used to fit a ground truth FA. To evaluate the generalizability of each network, they were tested on two external clinical datasets, not seen during training, and acquired on different scanners with different protocols, as previously done.

**Results:**

Using 4 or 7 DW volumes, it was possible to train DL networks to obtain FA maps with the same range of values as ground truth - map, only when using HCP test data; pathological sensitivity was lost when tested using the external clinical datasets: indeed in both cases, no consistent differences were found between patient groups. On the contrary, our “one-minute FA” did not suffer from the same problem.

**Conclusion:**

When developing DL networks for reduced acquisition times, the ability to generalize and to generate quantitative biomarkers that provide clinical sensitivity must be addressed.

## 1 Introduction

Diffusion weighted (DW) imaging is a non-invasive method that allows to reconstruct quantitative maps sensitive to the underlying architecture of the tissue: the microscopic random diffusion of water molecules is exploited to obtain information on the microstructure of the brain. Diffusion abnormalities can reflect, at the macroscopic level, changes in microscopic tissue organization (Le Bihan et al., 1991; Jones, 2011).

DW imaging has the major benefit of providing data that can be used to derive quantitative maps. From the simplest formalism used to describe water diffusion in tissue, i.e., the diffusion tensor (DT) (Basser et al., 1994; Pierpaoli et al., 1996), it is possible to calculate maps of fractional anisotropy (FA), an index that is highly sensitive to microstructural damage of brain tissue due to pathological processes (Alexander et al., 2007). Mathematically, 7 DW volumes must be acquired to fully characterize the DT: 6 DW measurements along noncollinear directions and 1 with no DW, i.e., *b*-value = 0 (Tournier et al., 2011). It has been noted that limiting the number of diffusion directions to 6 can introduce directional biases in DT metrics. Many studies have shown that to avoid this problem, it is necessary to increase the number of DW directions that also contribute to improving the signal-to-noise ratio of the obtained maps (Giannelli et al., 2010; Zhan et al., 2011; Lebel et al., 2012). However, increasing the number of acquired DW images inevitably increases acquisition times. Some studies, therefore, have investigated the possibility of reducing the number of DWs to obtain FA by deep learning (DL) methods (Li et al., 2019; Aliotta et al., 2021; Gaviraghi et al., 2022).

Aliotta et al. (2021) developed a specific network for assessing FA in gliomas, without testing it on other clinical datasets nor on datasets acquired on other scanners or with different acquisition protocols. Li et al. (2019) developed a network by training it only on healthy subjects and did not test it on clinical cases or on other datasets. In our previous work (Gaviraghi et al., 2022) we optimized a “one-minute FA” DL network, which has the architecture of a U-net (Ronneberger et al., 2015) and requires 10 DW input volumes to output the FA map. This network was able to give as output FA maps that retained the quality of the FA maps obtained with the high-resolution fully DW sampled human connectome project (HCP) data (which has 288 DW volumes) (Van Essen et al., 2012; WU-Minn Consortium Human Connectome Project, 2017) used for model training. Differently from the previously mentioned papers, we also tested the generalizability of the network and its sensitivity to pathology in independent temporal lobe epilepsy (TLE) and multiple sclerosis (MS) datasets, acquired with different protocols on different scanners, without the need for retraining.

Recent work such as that of Aliotta et al. (2021) reconstructed the FA map from 3 DW volumes plus one *b*-value equal to 0, but did not show whether such network can be applied to unseen data acquired with different protocols and in different pathological cases. Moreover, given that the DT needs 6 DW directions to be defined, one may question how generalized this method really is. A fundamental characteristic of our “one-minute FA” DL network was that it was generalizable, i.e., applicable to datasets other than the one used to train the network. By reducing to extreme situations the input DW volumes, here we wanted to assess whether it was possible to retain output quality, sensitivity to pathology and generalisability. Indeed, it is by ensuring preservation of this fundamental characteristic of a DL network, i.e., generalisability, where the network becomes applicable to other, unseen datasets, where clinical translation becomes possible.

For medical images, data fidelity is essential, indeed it is necessary to avoid that in the images reconstructed by the DL networks there are pathological characteristics in the absence of the pathology and, on the other hand, non-pathological characteristics where there is pathology (Gassenmaier et al., 2021). Therefore, the main aim of the present work was to determine the minimal number of DW volumes required to maintain the clinical sensitivity of the reconstructed FA maps, independently from the specific pathology. More in detail, our goal here was to obtain a network able to output an FA map with the lowest possible number of DW volumes required as input, but at the same time able to maintain the same characteristics of the ground truth (GT) FA calculated with all volumes. The overall objective was to find a compromise between reducing the acquisition time and maintaining the original characteristics of the FA maps in healthy and pathological test sets.

## 2 Materials and methods

### 2.1 Subjects

The studies involving human participants were reviewed and approved by NRES Committee London–City Road and Hampstead and the Local Ethic Committee of the IRCCS Mondino Foundation. The patients/participants provided their written informed consent to participate in this study.

This work we used three datasets with the following characteristics:

HCP dataset: Pre-processed data of 76 HCP healthy controls (HC) (43 women, 29.41 ± 3.62 years)^1^ (Van Essen et al., 2013), used to train the network.

Temporal lobe epilepsy (TLE) dataset: Retrospective dataset used to test the performance of the network. 84 subjects: 34 HCs (16 women, 31.97 ± 7.73 years), 21 TLE patients with the epileptogenic zone in the left hemisphere (LTLE; 13 women, 33.13 ± 11.28 years), and 29 TLE patients with the epileptogenic zone in the right hemisphere (RTLE; 17 women, 37.97 ± 9.86 years) (Gaviraghi et al., 2021).

Multiple sclerosis (MS) dataset: Retrospective dataset used to test the performance of the network. 123 subjects: 29 HCs (19 women, 34.58 ± 10.23 years), 18 patients with clinically isolated syndrome (CIS; 12 women, 49.01 ± 7.16 years), 63 patients with relapsing–remitting MS (RRMS; 48 women, 47 ± 7.58 years), and 13 patients with secondary progressive MS (SPMS; 9 women, 47.83 ± 7.79 years) (Brownlee et al., 2019).

### 2.2 MR acquisition and pre-processing

The acquisition protocols, for each dataset, are summarized below:

HCP dataset: Siemens 3T Connectome Skyra scanner with a dedicated gradient insert. Sequences included: DW spin-echo EPI sequence with TR = 5520 ms and TE = 89.5 ms, resolution = 1.25 mm^3^ × 1.25 mm^3^ × 1.25 mm^3^ and matrix size = 145 × 174 × 145, 288 DW volumes (18 with *b*-value *b* = 0 s/mm^2^ and 270 with *b* = 1000/2000/3000 s/mm^2^, i.e., 90 noncollinear DW directions for each *b*-value). 3D T1-w data with 0.7 mm^3^ × 0.7 mm^3^ × 0.7 mm^3^ resolution and co-registered to the DW data (to obtain a resolution of 1.25 mm^3^ × 1.25 mm^3^ × 1.25 mm^3^).

TLE dataset: Siemens 3T MAGNETOM Skyra scanner with standard gradients. DW spin-echo EPI sequence with TR = 8,400 ms and TE = 93 ms, resolution = 2.24 mm^3^ × 2.24 × 2.2 mm^3^, and matrix size = 100 × 100 × 96, 109 DW volumes (13 with *b*-value *b* = 0 s/mm^2^ and 96 with *b* = 1000/2000 s/mm^2^, i.e., 48 noncollinear DW directions for each *b*-value). 3D T1-w data with 1 mm^3^ × 1 mm^3^ × 1 mm^3^ resolution.

MS dataset: 3T Philips Achieva MRI scanner with 80 mT/m maximum gradient strength. DW spin-echo EPI with TR = 14,000 ms and TE = 82 ms, resolution = 2.286 mm^3^ × 2.286 mm^3^ × 2.5 mm^3^, and matrix size = 96 × 96 × 60, 60 DW volumes (7 with *b*-value *b* = 0 s/mm^2^ and 8/15/30 with *b* = 300/711/2000 s/mm^2^). 3D T1-w data with 1 mm^3^ resolution.

For the clinical datasets, TLE and MS, the pre-processing steps included denoising, Gibbs ringing artifact, EPI distortion, eddy current, and subject motion correction (Gaviraghi et al., 2022).

For each dataset, the FA used as GT was obtained by fitting the diffusion kurtosis model to all acquired DW data (i.e., using the maximum number of DW volumes available), to obtain greater accuracy than with the tensor fitting model (Veraart et al., 2011). This will be referred to as the STANDARD method for calculating FA as opposed to using the DL network trained for the purpose.

### 2.3 Data preparation

**FIGURE 1:**
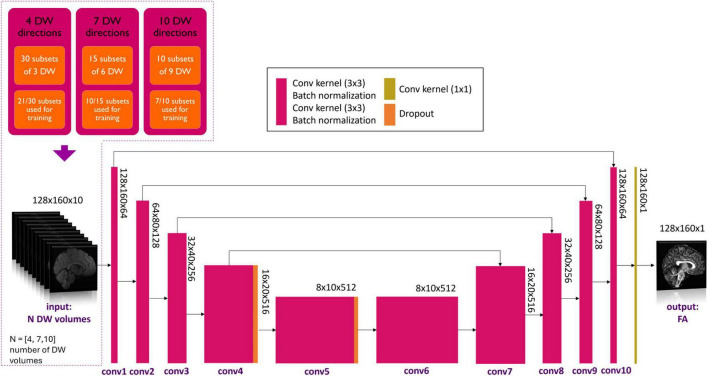
Details of the network input are shown in the top left-hand corner. Three experiments with different numbers of DW volumes as input (*N* = 4, 7 and 10) were considered. Training always used 7/10th of all subsets for each experiment. Network architecture is shown, with the color legend for the layers shown in the box (Conv, convolution). Adapted from Gaviraghi et al. (2022).

Each DL network was based on the U-net architecture (Figure 1). The training of each DL network was conducted using the hyperparameters defined in “one-minute FA” (Gaviraghi et al., 2022). As in the previous work, of the 76 healthy controls of the HCP dataset, 54 were used for the training set, 11 for the validation set and 11 for the test set. The only difference is the number of input DW images, thus the number of input channels of the network.

### 2.4 Training design

Several combinations of *N* input, namely *N* = 4, 7 and 10 DW volumes, were explored. A network was trained on each of these input data set that were divided into subsets. In previous work (Gaviraghi et al., 2022), different subsets of DW were tested to create a generalized network, i.e., that is less dependent on the encoding directions of the diffusion data used for training. By using 7 out of 10 subsets, the best performance was achieved. Each network, therefore, was trained on the HCP data and the number of DW input subsets used for training was set to be 7/10th of the total number of subsets possible for all combinations, so that the training sets had a similar amount of data (Figure 1). In each experiment, the Camino toolkit (Cook et al., 2005) was used to divide the DW volumes into subsets of *N* volumes with DW weighting equally distributed on the sphere.

In the case of *N* = 4 DW volumes, the 90 volumes with *b*-value equal to 1000 s/mm^2^ were divided into 30 subsets of 3 volumes each. The network was trained using 21 subsets of 3 DW volumes plus one b0.

In the case of *N* = 7 DW volumes, the 90 volumes with *b*-value equal to 1000 s/mm^2^ were divided into 15 subsets of 6 volumes each. The network was trained using 10 subsets of 6 DW volumes plus one b0 volume. From theory, 7 is the minimum number of noncollinear DW volumes required to estimate the diffusion tensor using the standard method.

In the case of *N* = 10 DW volumes, the 90 volumes with *b*-value equal to 1000 s/mm^2^ were divided into 10 subsets of 9 volumes each. The network was trained using 7 subsets of 9 DW volumes plus one b0.

Once the network parameters were set for each of the *N* input volumes, each network was applied to the HCP test subjects and clinical datasets i.e., TLE and MS.

### 2.5 Performance assessment

For each subject, white matter (WM) FA was calculated twice: with the STANDARD method using all volumes and with the network using a reduced set of volumes. For MS subjects, normal appearing WM (NAWM) was considered, i.e., the WM mask without lesions. To compare the WM FA calculated using the standard method with the WM FA calculated with the network for each subject, three different performance metrics were calculated: the root mean square error (RMSE), mean absolute error (MAE) and structural similarity index measure (SSIM) (Wang et al., 2004). We compared histograms of each single subject WM FA values obtained with the two methods. Heatscatters were plotted with the WM FA STANDARD values on the *x*-axis and the WM FA values of the network on the *y*-axis, and the R^2^ coefficient was calculated using the following formula:


$$R^{2}=1-\frac{\sum_{i=1}^{n}{\left(y_{i}-{\hat{y}}_{i}\right)}^{2}}{\sum_{i=1}^{n}{\left(y_{i}-{\bar{y}}_{i}\right)}^{2}}$$

where *n* = number of voxels that belong to the brain mask, *y_i_* is the desired output (GT FA) and ${\hat{y}}_{i}$ is the network output.

In addition, at the single-subject level, Bland–Altman plots were displayed, considering the FA of all WM voxels extracted from both maps, which allowed us to check the outliers distribution for each case. At group-level the values were compared using boxplots and the Mann–Whitney U-test (*p* < 0.05) was performed to assess group differences using either GT WM FA or the network WM FA. Bland–Altman plots, for the clinical datasets, were also performed at group-level, considering the average WM FA of each subject (Supplementary Figure 1).

## 3 Results

For each number of N DW inputs, FA was successfully obtained for all HCP subjects of the test dataset and for all subjects belonging to the TLE and MS datasets. Table 1 show three performance metrics (RMSE; MAE; SSIM) for each network, and for each dataset.

**TABLE 1:** For each metric [root mean square error RMSE, mean absolute error MAE, structural similarity index measure (SSIM)] the mean and standard deviation across all subjects is shown.

	HCP	TLE	MS
**RMSE**			
10 DW	0.046 ± 0.002	0.1 ± 0.005	0.1 ± 0.011
7 DW	0.05 ± 0.002	0.117 ± 0.023	0.113 ± 0.02
4 DW	0.069 ± 0.003	0.137 ± 0.021	0.139 ± 0.019
**MAE**			
10 DW	0.035 ± 0.002	0.079 ± 0.005	0.078 ± 0.009
7 DW	0.039 ± 0.002	0.088 ± 0.017	0.086 ± 0.014
4 DW	0.052 ± 0.002	0.104 ± 0.015	0.107 ± 0.014
**SSIM**			
10 DW	0.9 ± 0.008	0.743 ± 0.016	0.698 ± 0.05
7 DW	0.89 ± 0.009	0.645 ± 0.092	0.57 ± 0.096
4 DW	0.82 ± 0.014	0.544 ± 0.081	0.419 ± 0.073

Each column represents a dataset (HCP, TLE, MS) and each row represents a different network varying the number of diffusion weighted volumes as input (10, 7, 4 diffusion weighted volumes).

**FIGURE 2:**
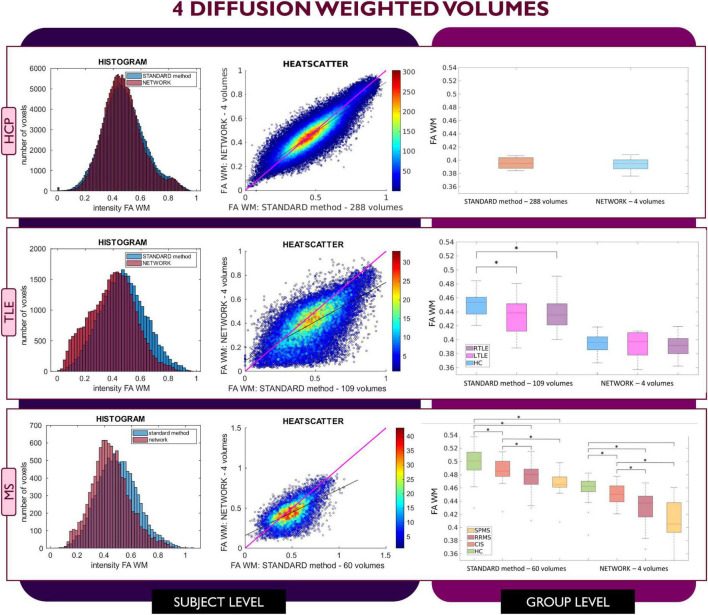
Comparison of the white matter (WM) fractional anisotropy (FA) for the experiment using 4 diffusion weighted (DW) volumes. Each row shows a different dataset, from top to bottom: human connectome project (HCP), temporal lobe epilepsy (TLE) and multiple sclerosis (MS). From left to right, columns show histograms, heatscatter plots and boxplots of WM FA (normal appearing WM—NAWM—for the MS case). Significant differences between clinical groups are indicated in the boxplots with an asterisk.

**FIGURE 3:**
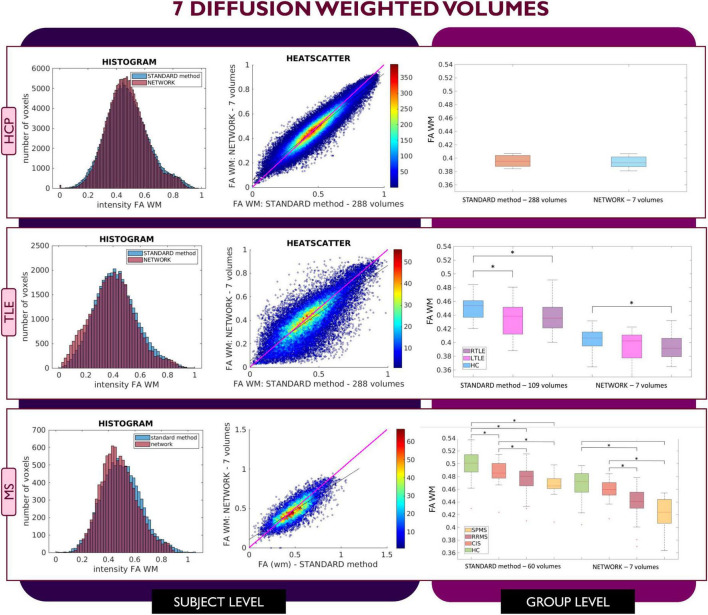
Comparison of the white matter (WM) fractional anisotropy (FA) for the experiment using 7 diffusion weighted (DW) volumes. Each row shows a different dataset, from top to bottom: human connectome project (HCP), temporal lobe epilepsy (TLE) and multiple sclerosis (MS). From left to right, columns show histograms, heatscatter plots and boxplots of WM FA (normal appearing WM—NAWM—for the MS case). Significant differences between clinical groups are indicated in the boxplots with an asterisk.

**FIGURE 4:**
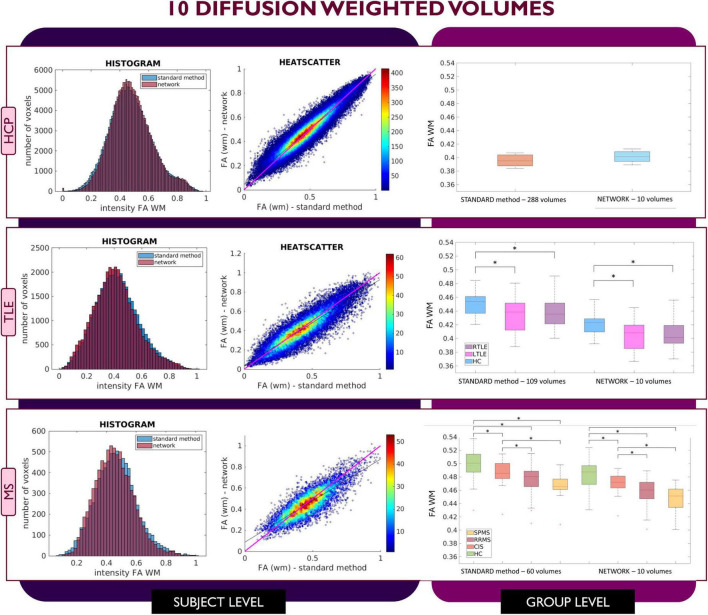
Comparison of the white matter (WM) fractional anisotropy (FA) for the experiment using 10 diffusion weighted (DW) volumes. Each row shows a different dataset, from top to bottom: human connectome project (HCP), temporal lobe epilepsy (TLE) and multiple sclerosis (MS). From left to right, columns show histograms, heatscatter plots and boxplots of WM FA (normal appearing WM—NAWM—for the MS case). Significant differences between clinical groups are indicated in the boxplots with an asterisk.

Figures 2–4 show plots for each experiment, i.e., with *N* = 4, 7 and 10 input DW volumes, respectively. In each figure, the first row refers to a representative HCP test subject, the second row to the TLE dataset and the last row to the MS dataset. Each figure for each dataset reports, from left to right, the histogram, heatscatter plot (single level analysis) and the boxplot (group level analysis), all reporting WM FA values obtained with each method. The statistically significant group differences are reported as asterisks (*p* < 0.05). The R^2^ coefficients are shown in Table 2.

**TABLE 2:** R^2^ coefficients are shown for a random subject for each dataset.

	4 DW	7 DW	10 DW
HCP	0.7818	0.8820	0.9062
TLE	0.6113	0.4557	0.8205
MS	0.3888	0.6611	0.7682

HCP, Human connectome project; TLE, temporal lobe epilepsy; MS, multiple sclerosis. Each column shows a different experiment i.e., 4, 7 and 10 diffusion weighted (DW) volumes.

When using 4 DW volumes (Figure 2) as input to the network, it is possible to appreciate that for the HCP test subjects the FA maps have similar characteristics to those calculated with the STANDARD method using all volumes. Conversely, the TLE dataset shows that statistical significance is lost between HC and LTLE and between HC and RTLE when using the network FA. For the MS dataset, between group differences are maintained, but NAWM FA values calculated with the network are much lower than with the STANDARD method.

Similar considerations can be made for FA values obtained with the 7 DW network (Figure 3) where for the HCP test subjects, FA maps have comparable characteristics to those calculated with the STANDARD method using all DW volumes. In contrast, for the TLE dataset, the statistically significant difference between HC and LTLE WM FA mean values was not reached when using WM FA values obtained using the network. For the MS dataset, the statistically significant difference between the HC and CIS was not reached when using WM FA values from the network.

This behavior was not detected in the case of network FA values obtained when inputting 10 DW volumes to the network (Figure 4); indeed, for all three datasets FA maps displayed similar characteristics as the FA calculated with the STANDARD method using all volumes and the clinical dataset displayed the same statistically significant differences (*p* < 0.05) between groups when using either the STANDARD method WM FA or the network WM FA.

**FIGURE 5:**
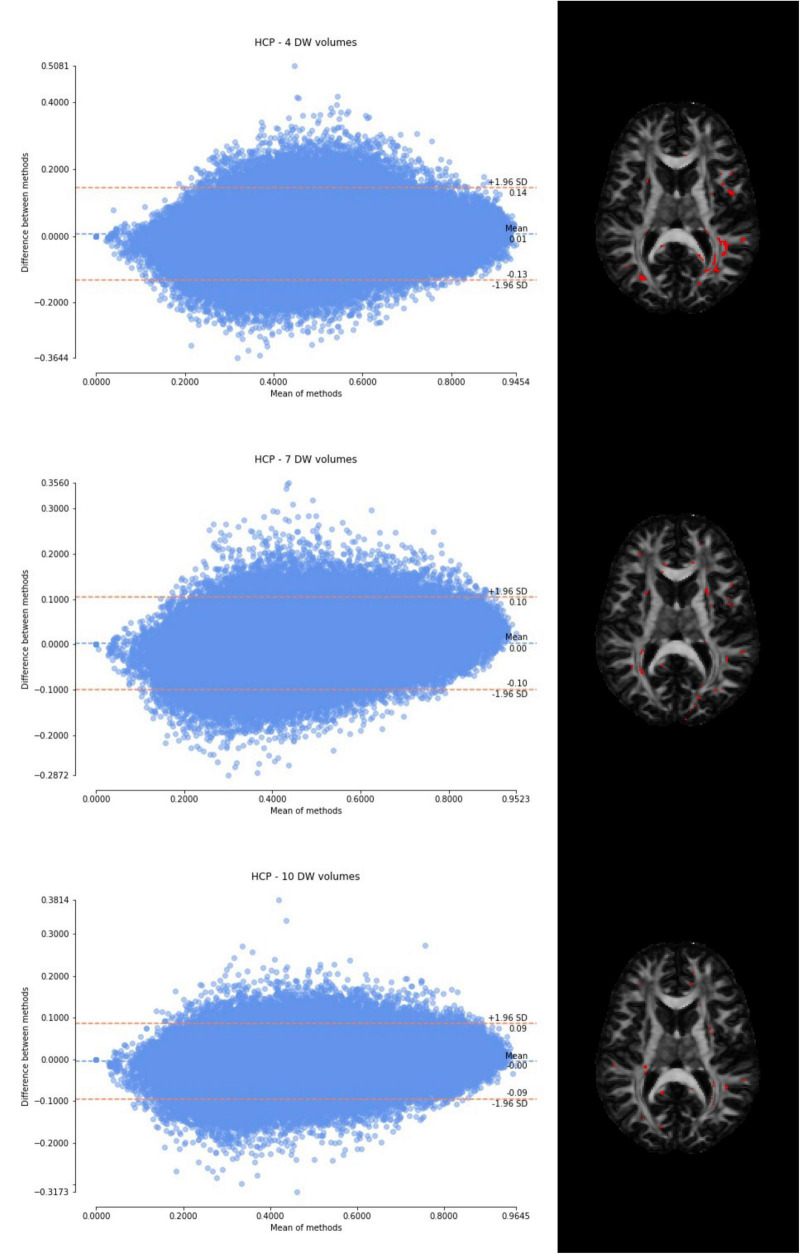
From top to bottom, for a randomly chosen HCP test subject, Bland–Altman plots of all WM FA voxels are shown, with increasing the number of diffusion-weighted (DW) volumes given in input to the deep learning network, i.e., from 4 to 10. On the right, an axial image of the brain shows the location of the outliers (red voxels).

**FIGURE 6:**
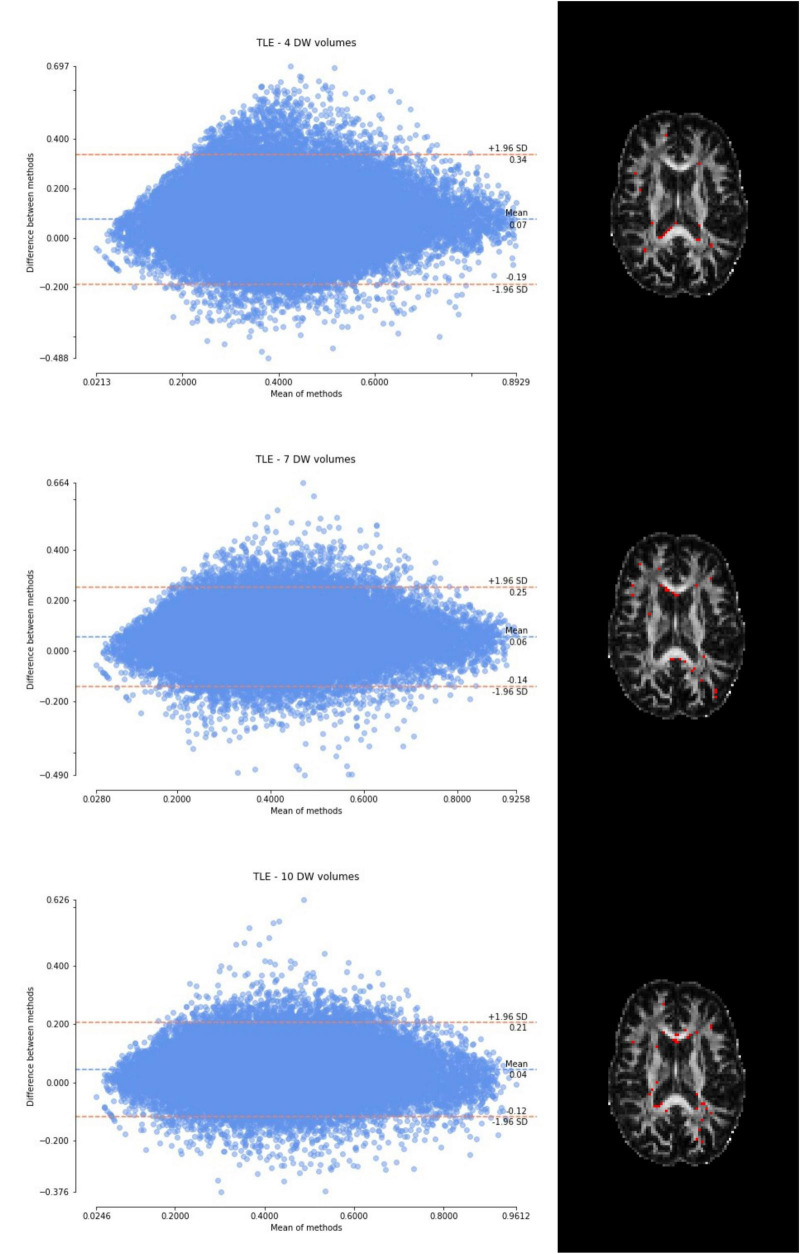
From top to bottom, for a randomly chosen temporal lobe epilepsy (TLE) subject, Bland–Altman plots of all WM FA voxels are shown, with increasing the number of diffusion-weighted (DW) volumes given in input to the deep learning network, i.e., from 4 to 10. On the right, an axial image of the brain shows the location of the outliers (red voxels).

**FIGURE 7:**
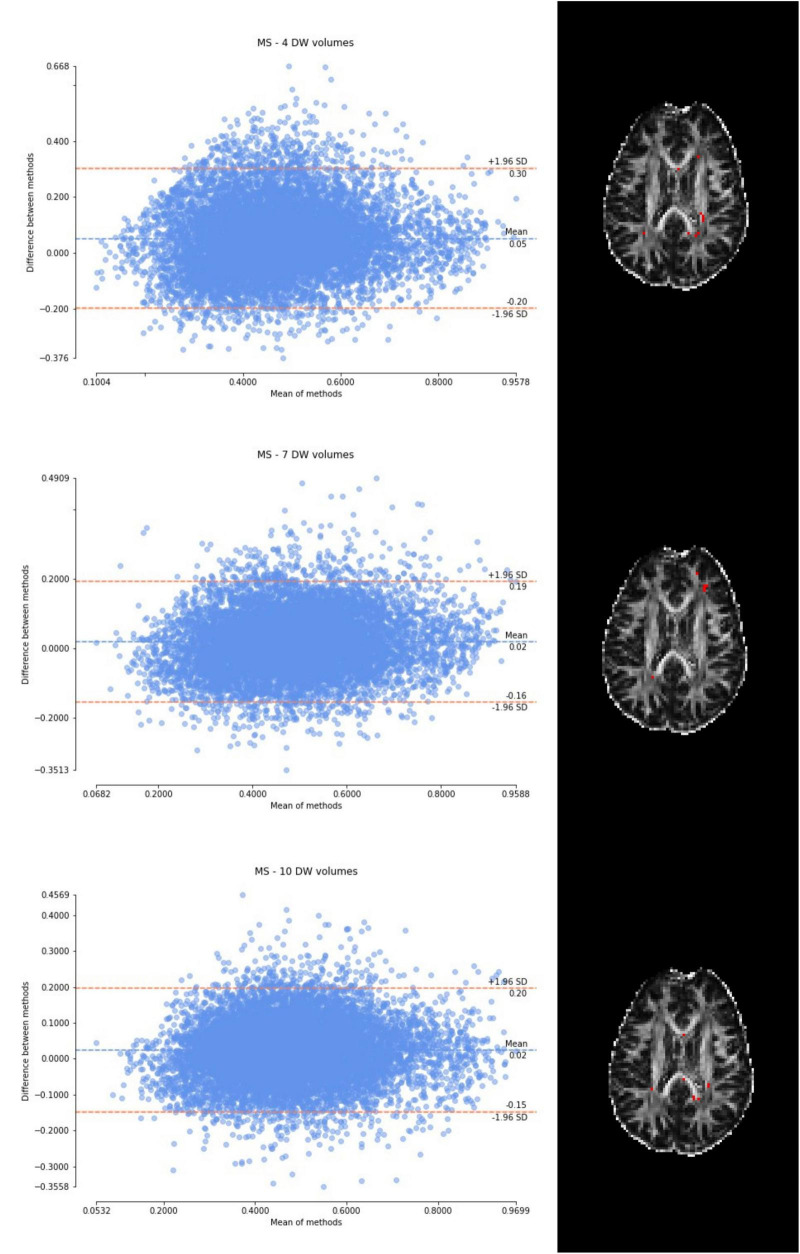
From top to bottom, for a randomly chosen multiple sclerosis (MS) subject, Bland–Altman plots of all WM FA voxels are shown, with increasing the number of diffusion-weighted (DW) volumes given in input to the deep learning network, i.e., from 4 to 10. On the right, an axial image of the brain shows the location of the outliers (red voxels).

Considering the Bland–Altman plots for each dataset (Figures 5–7), by increasing the number of DW volumes from 4 to 10, the number of outliers decreases. The outliers are almost all distributed at the interface between white matter and gray matter or between white matter and cerebrospinal fluid (CSF).

## 4 Discussion

As the number of input DW volumes decreased, performance worsened evaluated using each performance metric (Table 1). All networks (4 DW, 7 DW and 10 DW) performed well on the test HCP data demonstrating the ability of the network to learn the mapping between a very reduced DW input dataset and GT FA. Conversely, for the clinical datasets, the sensitivity to pathology was partially lost with extremely reduced input datasets to the networks, i.e., 4 and 7 DW volumes.

Indeed, when using the 4 DW network FA no statistical differences were detected between HC and TLE patients; statistically significant differences between HC and MS patients were still detected, but NAWM FA values were much lower than those calculated with the STANDARD method. Interestingly, the standard deviation of the network FA in the SPMS group was increased compared to the STANDARD one, possibly due to a greater level of tissue heterogeneity typical of this group, due to severe pathological damage, not fully captured by training the network on limited input volumes of healthy subjects. When using 7 DW input volumes to the network, a similar drawback was observed: indeed, in the TLE dataset, the network FA did not identify differences between HC and LTLE, and in the MS dataset, differences were not found between HC and CIS patients.

In the experiments with 4 and 7 DW input volumes, the same hyperparameters of the network calculated with 10 DW were used (Gaviraghi et al., 2022). The fact that the performance is excellent on the HCP test subjects suggests that retraining hyperparameters would not provide an improvement of performance on the validation of unseen clinical datasets.

On the other hand, when the network is trained on more than the minimum number of DW volumes required to define the diffusion tensor, clinical sensitivity is maintained as it was the case for the 10 DW network (our proposed “one-minute” FA network).

Varying the number of inputs volumes to 4 and 7 showed us how an extreme reduction of the input information passed to the network may affect the network capability of generalization. Our previously proposed “one-minute FA” network can, therefore, extract FA from a reduced set of 10 DW volumes, not only on test data with identical acquisition properties as the training data, but also on test data with different diffusion-encoding directions and, most importantly, on data acquired on different scanners, with different DW directions and different *b*-values (Gaviraghi et al., 2022).

The WM FA values obtained from the network, as can be seen from the boxplots, are always lower than in the standard method with all volumes, except in the case of the HCP test set. This could be due to the fact that only the HCP dataset was used for training the network, or it could be a partial volume effect with gray matter or even CSF due to the fact that the voxel size of the clinical datasets (> 2.2 mm) is larger than that of the HCP training data (1.25 mm) (hence reducing the number of voxel with high FA). Interestingly, our data shows that the number of outliers, i.e., the voxels that behave differently than the FA calculated with all data available, decreases as more DW volumes are used for the deep learning network. Moreover, the outliers seem to be distributed exactly in regions of greater partial volume effect (between WM and gray matter or CSF or between crossing fibers). This could be further tested by acquiring data with the same voxel resolution as the HCP data, but using a different scanner (Fujiwara et al., 2008).

In this work we did not change the resolution of the training dataset to match the clinical dataset because we wanted to assess whether the network could learn the non-linear relationship between the low and high quality scans and consequently be applied to any dataset, independently of the acquisition parameters, we believe that as FA is derived from the DT model, training the DL network on data with lower DW directions than the minimum required for its mathematical definition, it cannot capture in full signal changes caused by different underlying microstructure scenarios. Nevertheless, in future, to investigate how much the resolution impacts on the network’s performance, training could be performed again by resampling the diffusion images of the HCP dataset to different resolutions. In this way, it could be understood whether performance improves by training the network at the resolution of the clinical images.

In future work, different architectures could also be explored, such as CycleGAN (Zhu et al., 2017), to investigate whether the performance improves. In addition, as future work, the number of subjects used for training could be expanded, including also subjects with different pathologies to investigate whether the performance improves, without compromising generalizability.

In conclusion, here we investigated the dependency of DL network FA maps on the number of DW volumes used as input. With 4 or 7 DW volumes, clinical sensitivity of the network FA decreases compared to that of the GT FA. Reducing the data required as input to DL networks trained to obtain quantitative maps such as FA is an appealing proposal in term of scan time and cost/benefit evaluations, but reducing the input data to extreme cases can have a detrimental effect on obtaining a network capable of generalization. When developing DL methods for clinical adoption it is important to reach a good compromise between data acquisition time, generalizability, and clinical sensitivity of the network output.

## Data availability statement

The original contributions presented in this study are included in the article/Supplementary material, further inquiries can be directed to the corresponding author.

## Ethics statement

The studies involving humans were approved by the University College London IRCCS Mondino Foundation. The studies were conducted in accordance with the local legislation and institutional requirements. The participants provided their written informed consent to participate in this study.

## Author contributions

MG: Conceptualization, Formal analysis, Investigation, Methodology, Software, Validation, Visualization, Writing – original draft, Writing – review & editing. AR: Conceptualization, Methodology, Writing – review & editing. FuP: Data curation, Resources, Writing – review & editing. WB: Data curation, Resources, Writing – review & editing. PV: Resources, Writing – review & editing. FeP: Conceptualization, Data curation, Writing – review & editing. BK: Conceptualization, Data curation, Supervision, Writing – review & editing. CGW-K: Conceptualization, Funding acquisition, Methodology, Resources, Supervision, Writing – original draft, Writing – review & editing.

## Footnotes

1
http://db.humanconnectome.org
