# Supplementary material for: Finding the limits of deep learning clinical sensitivity with fractional anisotropy (FA) microstructure maps
Source: Front Neuroinform. 2024 Jun 12;18:1415085. doi: 10.3389/fninf.2024.1415085 (PMC11199892; doi:10.3389/fninf.2024.1415085)
Supplement: Supplementary file 1 [file Table_1.DOCX]

**Supplementary material**

Bland-Altman plots are performed to assess the agreement between white matter fractional anisotropy (FA) maps calculated with two different methods (standard and network) by plotting the differences against their means. Bland-Altman plots were calculated for the two clinical datasets, i.e., temporal lobe epilepy (TLE) and multiple sclerosis (MS), and for each number of diffusion weighted (DW) volumes in input (i.e. 4,7,10).

As the number of input DW volumes decreases, the mean difference and variance increase. Consequently, increasing variance can partially explain the decrease in clinical sensitivity.


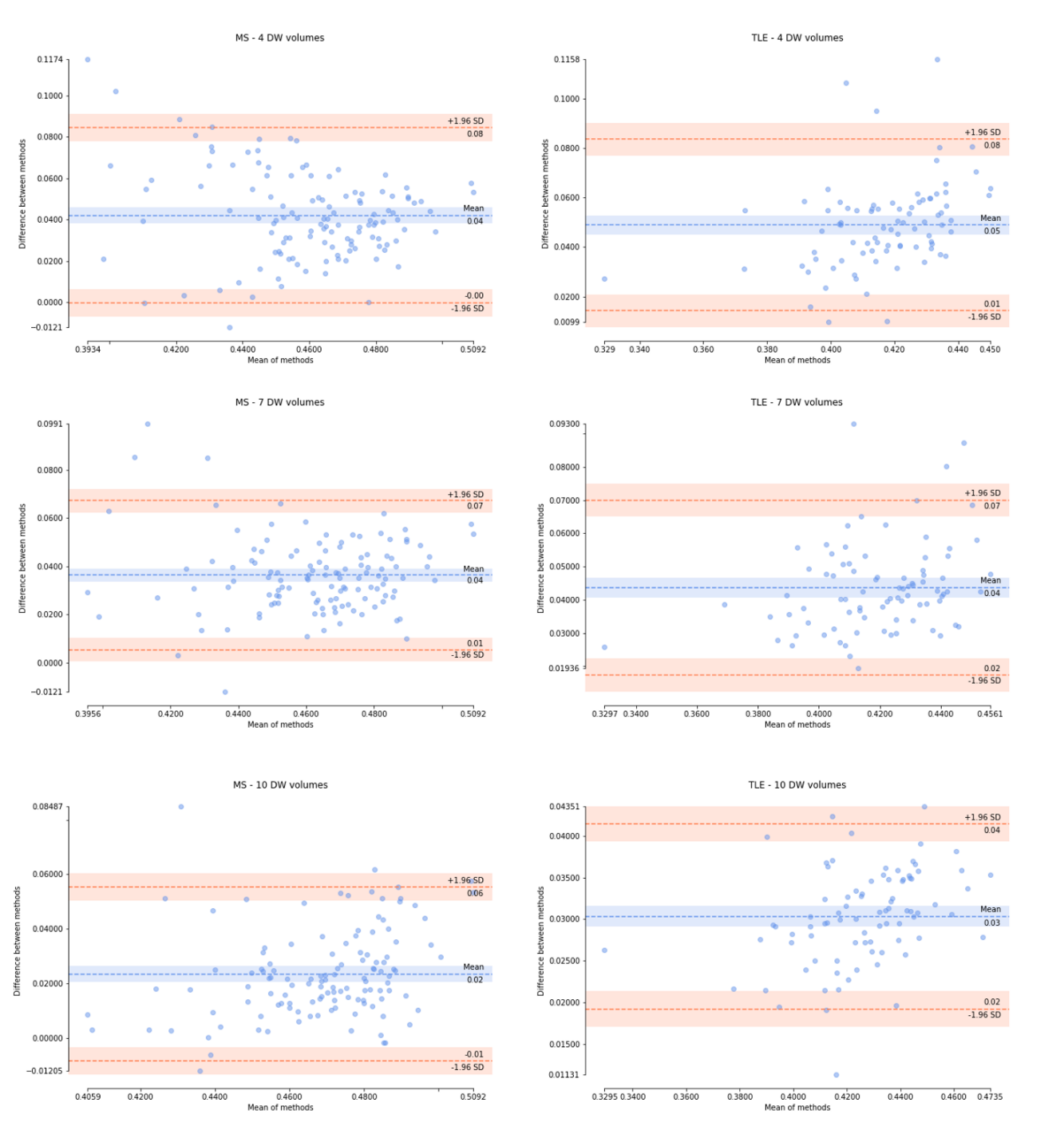


**Figure S1**: From top to bottom, Bland–Altman plots increasing the number of diffusion weighted volumes from 4 to 10. Each blue dot corresponds to a subject. The blue line indicates the mean of the difference between the white matter fractional anisotropy STANDARD method and NETWORK. The red lines indicate the limits of agreement (average difference ± 1.96 standard deviation of the difference). Temporal lobe epilepsy is reported on the first column, while multiple sclerosis is reported on the second column.
